# Effect of Temperature Control and Rotational and Traverse Speeds on the Mechanical Properties of Friction Stir-Welded Polypropylene Plates

**DOI:** 10.3390/polym16223110

**Published:** 2024-11-05

**Authors:** Miguelangel Balaguera, Habib R. Zambrano, Ramiro J. Chamorro Coneo, Juan Felipe Santa Marín, Jimy Unfried-Silgado

**Affiliations:** 1 Departamento de Ingeniería Mecánica, Grupo GIMYP, Universidad del Norte, Km 5 vía Puerto Colombia, Barranquilla 081007, Colombia; miguelangelb@uninorte.edu.co; 2 School of Mechanical Engineering, University of Campinas, Rua Mendeleyev, 200, Cidade Univesitaria Zeferino Vaz, Campinas 13083-840, SP, Brazil; rconeo@unicamp.br; 3 Departamento de Ingeniería Mecaníca, Universidad Nacional de Colombia-Sede Medellín, Carrera 64C No. 63–120, Medellín 050034, Colombia; jfsanta@unal.edu.co; 4 Departamento de Ingeniería Mecánica, Grupo ICT, Universidad de Córdoba, Montería 230002, Colombia; jimyunfried@correo.unicordoba.edu.co

**Keywords:** FSW in polypropylene, welding temperature control, crystallinity, polypropylene, polymer welding, processing parameters

## Abstract

In the present study, the effects of temperature and rotational and traverse speeds on the mechanical properties of polypropylene joints that are welded by friction stir welding using a non-rotational shoulder and a heat-assisted welding process is investigated. Tensile properties, microhardness measurements, microscopy observations, and thermal analysis are carried out in the present research to evaluate the effect of the welding parameters on the mechanical properties of welded joints. The experiments are conducted and analyzed by means of a central composite design using an analysis of variance (ANOVA). Variations in pre-heating temperature from 60 °C to 80 °C, rotational speed from 800 to 1500 rpm, and traverse speed from 20 mm/min to 100 mm/min are made for observations. A remarkable joint efficiency of 94% is achieved with joints that are free of discontinuities and defects. The fractured surfaces are observed to identify ductile and brittle zones. The crystallinity is measured, and a correlation between crystallinity and joint strength is discussed. The sample with highest efficiency shows 65% crystallinity and a ratio of 37.9% of ductile zone–total fractured area.

## 1. Introduction

**Figure 1:**
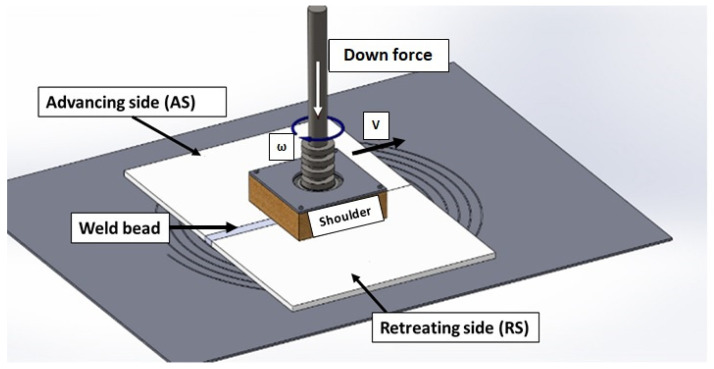
FSW parameters and main parts of the welding tool.

Friction stir welding (FSW) is a modern joining method that has been successfully used in lightweight metallic alloys such as aluminum, copper, and some low-alloy high strength steels. Recently, FSW applications have been extended to thermoplastics due to the advantages of this joining technology such as non-preparation, low process time, low machine/tool consumable cost, low temperature, high joint quality, low distortion, and greater material strength [1,2]. However, the implementation of this welding method in thermoplastics is still a challenge. FSW is performed using a rotational (non-consumable) tool consisting of a specially designed shoulder and pin. In general, the rotating tool penetrates into the two workpieces to weld, spinning at a selected rotational speed (*w*) and moving forward at a specific travel velocity (*v*). Therefore, considerable frictional heating is generated, causing strain softening in the material and creating a material flow to mix and join the components that are being welded, as shown in Figure 1. In FSW, the shoulder and pin are key tool parts which are in contact with the base material (Bm) during the welding process. The shoulder and pin also extrude and forge the material that is been welded [3,4]. Welding parameters such as *w*, *v*, tool axial force, welding temperature (*T*), and applied torque affect significantly the final mechanical properties of the FSW joint [5].

Polypropylene (PP) is one of the most versatile thermoplastics, and it is widely used for manufacturing laboratory equipment, plastic containers, pipe coatings for the oil and gas industry, auto parts, in the textile industry, and, more recently, in the hydrogen storage industry [6]. The main advantages of PP include low manufacturing cost, superior electrical, chemical and heat resistances, light weight, high resistance to flexing stress, good fatigue properties, high impact energy absorption, and excellent thermal stability [7]. In order to weld PP, several welding methods suitable for thermoplastics are available, such as melt fusion, hot gas (conduction), resistance, laser, induction (radiation), ultrasonic, and adhesive bonding [2,8,9]. However, these methods are not efficient due to the generation of imperfections, degradation of the Bm during the welding process, and the reduction of ductility in thermoplastics [2,10]. Therefore, a welding method such as FSW is highly competitive because the procedure offers superior joint quality, does not involve melting Bm, and is environmentally friendly. This innovative welding technique (FSW) offers a suitable alternative for high-quality joints in PP. However, in order to reduce the possibility of generating imperfections and defects in FSW joints for polymers, the temperature before and during the wielding process must be controlled. Therefore, the parameters that affect Bm temperature, such as tool velocity (*w* and *v*), axial force, and applied torque, must be studied to minimize the generation of defects and discontinuities [11,12,13,14,15]. Moreover, some authors suggest (for polymers) using a secondary heat source to obtain a better control of Bm temperature and to optimize the joint quality [16,17]. Other authors, like Romero et al. [18], suggest using a stationary shoulder to improve the frictional heating generated during the welding process. Regarding the FSW parameters for high-density polyethylene (HDPE), Romero et al. [18] establishes that the mechanical properties and crystallinity are significantly affected if *w* is varied in the range 846–1036 rpm, whereas regarding *v*, in the range 14–25 mm/min, the joint properties are slightly affected. Testing different *w* and *v* values during the FSW procedure, Romero et al., reports a maximum joint efficiency (*ξ*) of 90%, which is the quotient (in percentage) of the ultimate tensile strength (*S*_ut_) of the FSW joint divided by *S*_ut_ of the Bm. However, the effect of controlling the temperature during the FSW process is not investigated in [18]. Kiss and Czigany [19] have also investigated the effect of *w*, *v*, and tool geometry on the joint resistance in PP sheets. These authors also analyze crystallinity by using differential scanning calorimetry (DSC). Kiss and Czigany, utilizing commercial milling tools with different groove slopes and inverting the rotational direction to avoid milling, have demonstrated that tool geometry is a relevant parameter which has a crucial impact on the final FSW joint properties [19]. They also report a reduction of 50% in the *S*_ut_ of the Bm, a maximum *ξ* of 78%, and an evident change in the mechanical behavior of the PP from ductile to brittle. However, the results show that FSW is applicable to PP, but more research work is necessary to improve *ξ*.

FSW for thermoplastics still faces some challenges due to insufficient heat generated by means of friction (between tool and Bm) and plastic deformation (during the stirring of the welded material). Usually, heat produced during welding is not enough to obtain a proper FSW joint in PP. Azarsa and Mostafapour [20], using plates of HDPE and integrating a heating source into the tool shoe, improve *ξ*, reporting a maximum flexural strength of 95.7% in comparison with the respective Bm strength. These results are obtained at the highest and lowest *w* and *v* (*w* = 1400 rpm and *v* = 25 mm/min), respectively, and at a shoe temperature of 110 °C. The results in [20] suggest that when providing supplementary heat during the FSW procedure, *ξ* increases significantly in comparison with the classic FSW process. The aim of the present work is to assess the effect of the most relevant parameters, viz. *w*, *v*, and *T*, on the mechanical properties of FSW joints in PP using a tool with a stationary shoulder and a secondary heat source system to provide preheating to the FSW process. This preheating control system warms the FSW joint from the bottom. In addition, changes in crystallinity are also investigated, as well as the possible correlation between crystallinity and *ξ*.

The main contribution of this study is the use of a secondary heat source system to provide preheating during the FSW process and the evaluation of parameters such as joint efficiency, crystallinity, and ductile zone–total fractured ratio.

## 2. Materials and Methods

### 2.1. Polypropylene Samples

Rectangular samples of 185 mm × 135 mm are cut out from PP plates with 5 mm thickness. The plates are acquired directly from the manufacturer, and they are made of non-recycled PP. Table 1 shows the mechanical and physical properties of PP, including melting temperature *T*_m_ and glass transition temperature *T*_g_. Before applying the FSW procedure, extrusion direction is established to carry out the welded joints perpendicular to this direction.

### 2.2. Welding Tool Configuration

**Figure 2:**
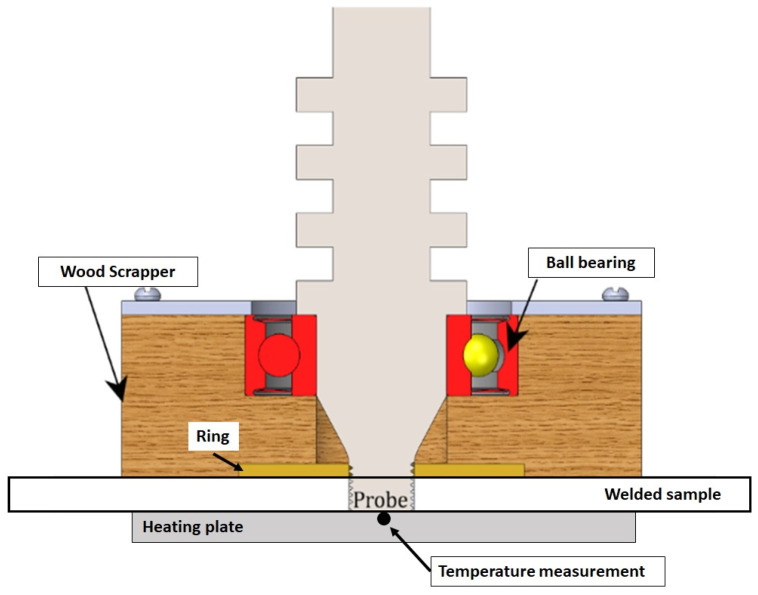
Schematic configuration of the FSW tool.

In the present investigation, FSW is performed by using a non-rotational shoulder tool made of AISI H13 steel. This type of FSW tool reduces the heat transfer between the tool surface and the stirred zone along the weld line. The tool configuration consists of a monolithic non-shoulder with a cylindrical threaded pin at the tip of the steel tool, a ball bearing, a square-shaped pinewood scraper, and a support ring-wing made of bronze, as shown schematically in Figure 2.

### 2.3. Preheating Control System and Temperature Monitoring

Temperature control is one of the parameters most relevant for the present research work, as some investigations suggest that controlling the temperature during the welding process has a significant effect on the mechanical properties of the FSW joints [21]. Therefore, a preheating control system is incorporated into the FSW process, which consists of a set of resistors that control the temperature through a current limiter. In addition, the temperature at the PP plates is directly monitored using a thermocouple.

### 2.4. Experiment Setup and CNC Equipment

**Figure 3:**
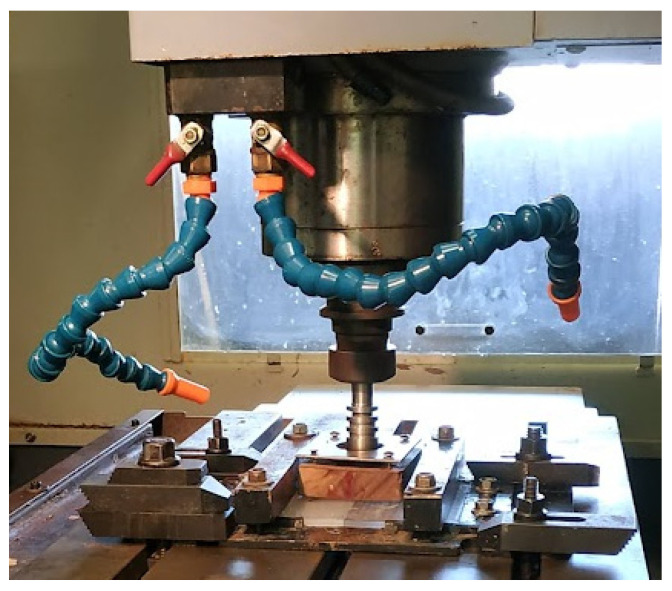
FSW tool and PP plates attached to the bench of the milling machine by means of a homemade support.

To perform the welds, the FSW tool is assembled on a conventional CNC milling machine (Leadwell^®^ V30 with a Fanuc^®^ system), and workpieces are attached to the bench of the milling machine using a homemade support which keeps the PP plates aligned during the welding process. The welding machine configuration is shown in Figure 3. Welding parameters (*w* and *v*) and PP temperature are controlled by the CNC milling machine and the preheating control system, respectively.

### 2.5. Process Parameters Selection

During the FSW process, the selected *w* and *v* for the FSW tool are varied in the range 800–1500 rpm and 20–100 mm/min, respectively. *w* and *v* are chosen according to values suggested in [22,23]. Regarding the preheating control system, the set temperature for each weld is selected in the range 53–86 °C, which is the temperature range where the highest *ξ* are obtained according to [6]. Assisted heating non-rotational shoulder tools with cylindrical taper pins used in FSW for polymer technologically offer excellent joint efficiency and surface finish, high material mixing quality, low probability of root and internal defects formation, and are very easy to implement [6].

### 2.6. Experimental Design

In this study, the central composite design (CCD) is used, which is usually applied for fitting a second-order model. Generally, the CCD involves running a 2*^k^* factorial experiment, where 2 represents the number of levels and *k* the number of factors. The CCD also has additional test points, viz. 2*k* star points runs, and center runs [24]. The center points set of experimental runs are the medians of the values used in the factorial part; to increase the experiment precision, this stage is frequently replicated. The star points are experimental runs with the same configuration as the center points, except for one factor, which can have both values inside and outside of the two factorial levels range median [24]. This is how all the factors are modified.

### 2.7. Experiments and Samples Preparation

The FSW joints are carried in accordance with a full-factorial CCD. The CCD has different levels for *w*, *v*, and *T*. Table 2 shows the levels for the parameters, which are controlled and monitored during the FSW process. The welded plates are used to machine out 18 tensile test specimens that are tested to assess mechanical properties of the FSW joints, thus evaluating the effect of welding parameters on the properties. The tensile specimens are machined out by means of the same CNC milling machine that is used for performing the welds. Tensile specimens are machined under low cutting velocity and constant flow of coolant to avoid affecting the microstructure. The tensile specimens fulfill the ASTM D638-14 standard [25]. The tensile tests are carried out in an MTS servohydraulic testing machine model 370 with load capacity of 50 kN. To contrast the results, tensile specimens are also machined from the Bm. The specimens are cut out perpendicular to the extrusion direction.

### 2.8. Microhardness Measurements

**Figure 4:**
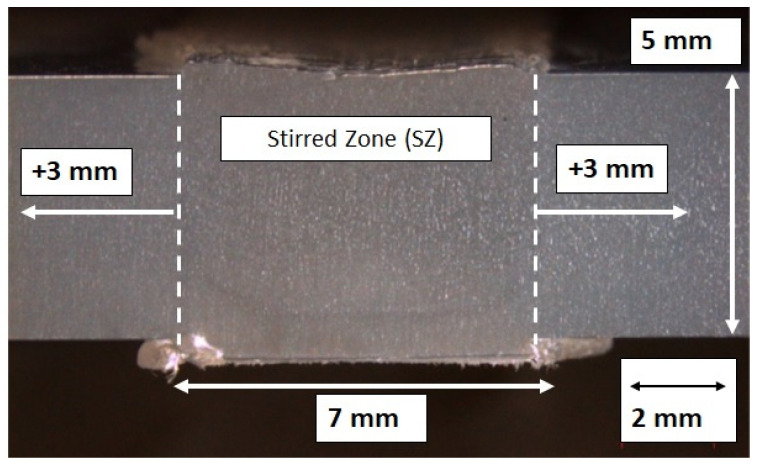
Microindentation area.

Microhardness measurements are performed along 13 mm of the specimen cross-section within the welded material, as shown in Figure 4. The microindentations are performed within the stirred zone (SZ), which has an average length of 7 mm, and 3 mm beyond the SZ on both sides, see Figure 4. The measurements are carried out using a Microhardness Akashi Testing Machine (MVK-H0 series), and the procedure fulfills the ASTM E384-17 standard [26].

### 2.9. Differential Scanning Calorimetry Analysis and Fractography

Glass transition temperature and crystallinity are analyzed by means of a differential scanning calorimetry analysis (DSC), which is performed using a Simultaneous Thermal Analyzer TGA/DSC (SDT-Q600, TA Instruments, New Castle, DE, USA). For the DSC, the samples are heat treated from room temperature to 800 °C, using a heating ramp of 10 °C/min and a controlled nitrogen atmosphere. The specimens with the lowest and highest *ξ* (viz., specimens 1 and 14) are evaluated by cutting out small samples from the SZ. Thus, crystallinity is assessed using the methodology suggested in [27]. In addition, a Scanning Electron Microscope (SEM) is also utilized to observe, in detail, the fractured zone of the samples.

## 3. Results

### 3.1. Tensile Test Results

Tensile test results for each FSW setup are reported in Table 3. Aiming to compute *ξ*, the material strength of the Bm is also measured, obtaining a *S*_ut_ = 35.5 MPa, where *ξ* is defined as the percentage ratio between *S*_ut_ for the FSW specimen and *S*_ut_ for BM. High plasticity and ductile behavior are evidenced during the tensile tests for Bm, as expected for PP [28,29]. On the other hand, the FSW samples exhibit a quasi-brittle fracture without yielding. Specimen 14 shows the highest *ξ* for test conditions: *w* = 1500 rpm, *v* = 40 mm/min, and *T* = 80 °C; specimens 1, 4, 6, 7, 9, 11, 16, and 18 yield moderate *ξ* up to 70%.

### 3.2. Analysis of Variance

The analysis of variance (ANOVA) is utilized to determine the significance level of the FSW parameters and their combinations in the current experimental work. The results of applying ANOVA on the tensile strength of the FSW welds are summarized in Table 4. Due to *p*-value < 0.05, it is observed the FSW parameters (that are studied in the present analysis) significantly affect the mechanical properties of the FSW joints. In order to establish significant differences among these parameters, a Least Significant Difference (LSD) test is used [30]. The results suggest that when using the highest values of *w* and *T* (viz. 1500 rpm and 80 °C, respectively) within the respective selected range, mechanical properties are significantly improved. On the other hand, high *v* values have a negative impact on *S*_ut_ for FSW joints. Therefore, the highest *S*_ut_ is obtained using the lowest *v* (viz. 40 mm/min), see Table 3.

### 3.3. Microhardness Profiles

**Figure 5:**
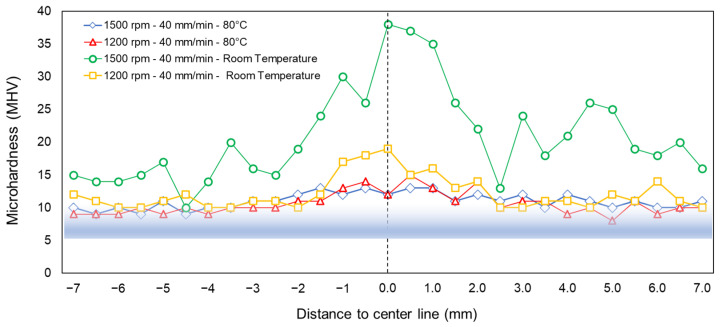
Microhardness profiles for different experimental conditions.

The temperature effect on the welded material is assessed by means of the microhardness profiles of the welded zone. Microhardness measurements are performed on samples 13 and 14, which report the best *ξ* values. The results are compared with microhardness profiles of specimens that are welded without heating control under the same *w* and *v*. Figure 5 shows microhardness profiles for the aforementioned specimens. The results suggest that the use of a heating control system during the FSW process significantly reduces the microhardness at the SZ in comparison with samples that are welded without heating control. The microhardness profiles show that the measurements for the Bm vary between 5 to 10 MHV. However, for samples that are welded under heating control at *T* = 80 °C, measurements vary between 9 to 14 MHV, while for samples that are welded without heating control, the results reach 38 MHV for *w* = 1500 rpm and *v* = 40 mm/min and 19 MHV for *w* = 1200 rpm and *v* = 40 mm/min. Therefore, using a heating control system, the microhardness is reduced by 50% comparing peak values.

### 3.4. Optical Microscopy Analysis for FSW Joints

**Figure 6:**
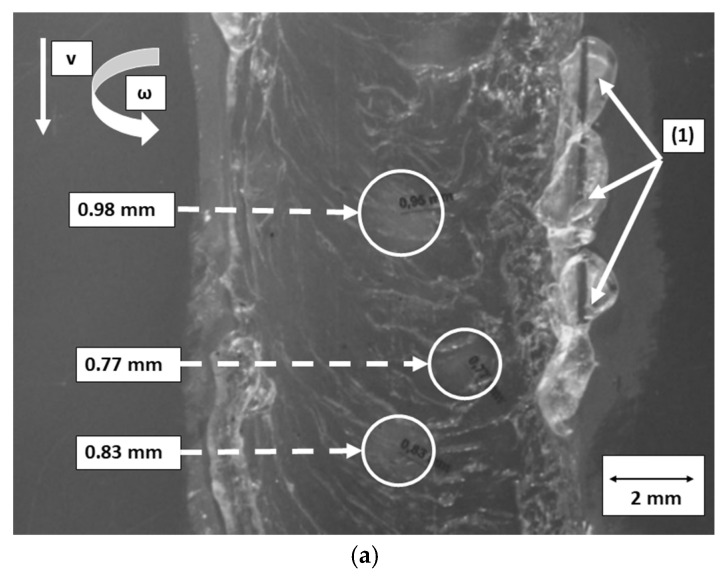

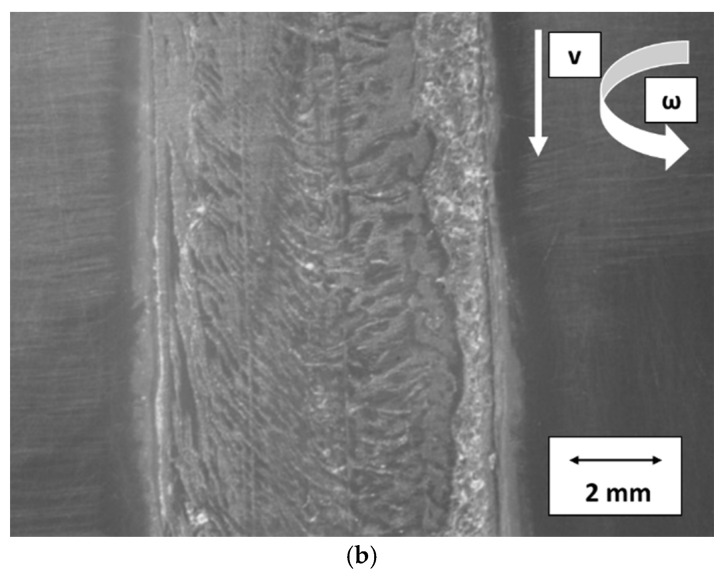
FSW zone for specimens (**a**) without using the preheating control system and (**b**) using the preheating control system at *T* = 80 °C.

Observing FSW zones of specimens that are welded without heating control, voids and imperfections are easy to recognize within the stir zone, as shown in Figure 6a. Void diameters are in the range 0.3–0.95 mm. In addition, a lack of warming is also observed along the welding path (which is indicated as (1) in Figure 6a). These imperfections reduce the transverse area that bears the loads, and they also act as stress raisers. Therefore, mechanical resistance of the FSW joint is affected, especially because the material behavior in the SZ is mainly brittle. However, when the specimens are welded using the preheating control system, small voids and cavities are not observable at the FSW joint surface. Consequently, surface roughness of the FSW joint is smoother, and a more homogeneous surface joint is obtained, as shown in Figure 6b.

### 3.5. Fractography of the FSW Joints Using DSC and SEM

**Figure 7:**
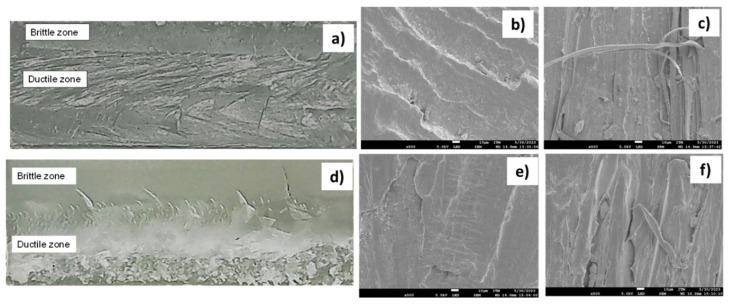
Fractured surfaces after tensile tests for Specimen 1 (**a**–**c**) and Specimen 14 (**d**–**f**).

The fracture surface of the FSW specimens is analyzed after the respective tensile tests. Two different zones are identified, viz. (i) a flat and dull zone and (ii) a fibrous and rough zone. The flat zone suggests brittle behavior during the fracture of the specimen, and the fibrous zone is related to a ductile mechanical response. Figure 7a–f show both zones, the brittle and ductile zones, for specimens 1 and 14, which are observed by means of a stereomicroscope and SEM. The brittle zone is typically composed of amorphous brittle glassy polymers (see Figure 7b,d). The main characteristic of this zone is the presence of crazes, which are often visible to the naked eye and look like a macroscopic crack (see Figure 7e) [31]. However, the ductile zone (DZ) exhibits elongated fibers, as shown in Figure 7c,f. The ratio of DZ to total fractured area (FA) is summarized in Table 5. A greater value of DZ/FA ratio evidences more ductility during the tensile test. This parameter (DZ/FA) is relevant because the resistance of amorphous polymers is usually higher than crystalline polymers [32]. In addition, Table 5 shows the base PP crystallinity, which is a parameter directly related to the polymer brittleness, stiffness, and optical properties. The reference value represents the heat of melting for a polymer that is 100% crystalline, and it is established as 207.1 in [33].

## 4. Discussion

### 4.1. Mechanical and Physical Properties

Regarding virgin PP properties, crystallinity (i.e., chains’ molecular order in the polymer structure) increases by more than 75% when the FSW process with heating control is applied [34]. For the test conditions that are evaluated in the present work, tensile test and microhardness results exhibit the highest values compared to results reported in other investigations for the same welded thermoplastic using heating control, i.e., [35,36]. Analyzing tensile test results and microhardness profiles for the different specimens, it is found that the highest *ξ* is obtained for the FSW specimens with microhardness profiles similar to the Bm hardness (see Table 3 and Figure 5). Moreover, the maximum *ξ* is observed in the sample with a minimum percentage of ductile zone, which is indicative of higher brittleness in the welding joint [37]. This result is also consistent with crystallinity measurements, which show a lower value for the sample with lower ductile zone percentage (as shown in Table 5). Normally, an increase in DZ is associated with a higher value in crystallinity [32,38].

### 4.2. Thermal History and FSW Joint Efficiency

Thermal history is a relevant parameter used in FSW studies when it is not possible to measure the applied torque, local temperature, and other variables required to establish heat input value. The heat pseudo index (*W*) aims to qualitatively determine the influence of the process parameters on heat generation [4]. *W* is defined as follows:

*W* = *w*^2^/*v* (1)

**Figure 8:**
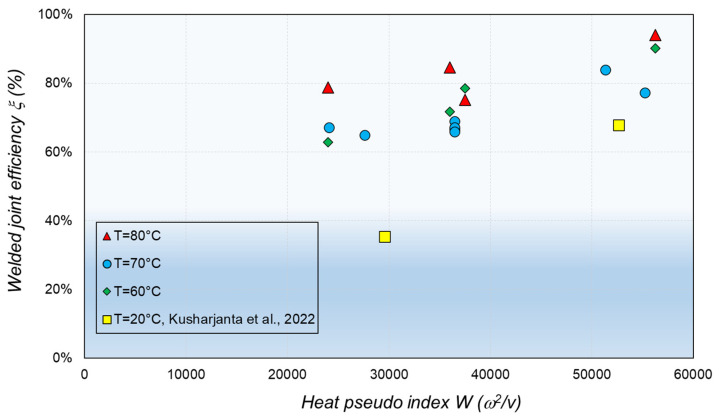
Effect of *W* on *S*_ut_ for FSW specimens [38].

**Figure 9:**
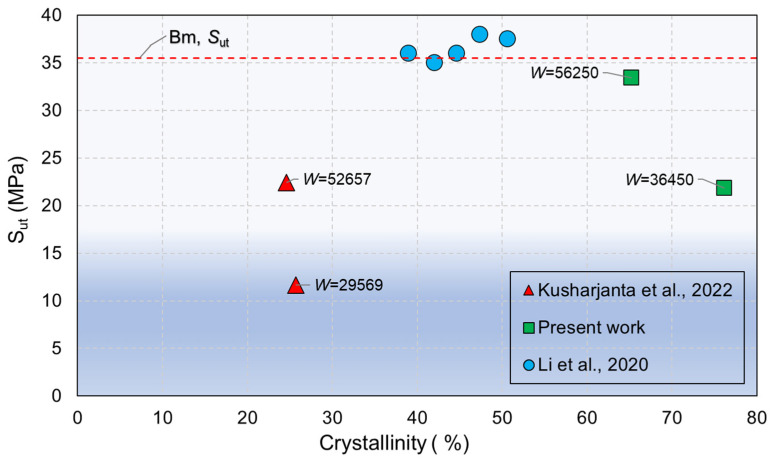
Effect of crystallinity on *S*_ut_ for FSW specimens [38,39].

*W* for all the FSW specimens are included in Table 3. The effect of *W* on *ξ* is evaluated in Figure 8. To contrast the results, data from [39] are also plotted in Figure 8. As seen in Figure 8, W for Sample 14 is nearly the double that for Sample 1; therefore, the heat generated for welding Sample 14 is nearly double, making it the example that exhibits the highest *ξ*. It is important to point out that the ductile and brittle zones display the same pattern on the fracture surface that is observed in Figure 7, which is consistent with the heat source location on the bottom of the welded samples. The results in Figure 8 suggest that the combination of the heat generated by the FSW process, and the heat provided by the heating control system significantly improves *ξ*. However, even if the precise selection of *W* can improve *ξ*, no use of a heating control system leads to a poor *ξ*. Regarding crystallinity, to analyze the correlation between this property and the FSW joint resistance, crystallinity vs. *S*_ut_ is plotted in Figure 9. Data published in [38,39] are included in Figure 9 to contrast the results. As seen in Figure 9, applying a lower *W* leads to a higher crystallinity in the stir region. However, reducing crystallinity of the PP located in the stir region during the FSW process yields higher *S*_ut_, as seen in Figure 9. On the other hand, Figure 9 shows results from [39], which represent the effect of crystallinity on the *S*_ut_ of plates made of PP that have not been subjected to FSW or any other manufacturing process.

### 4.3. Effects of Welding Parameters on Joint Efficiency

**Figure 10:**
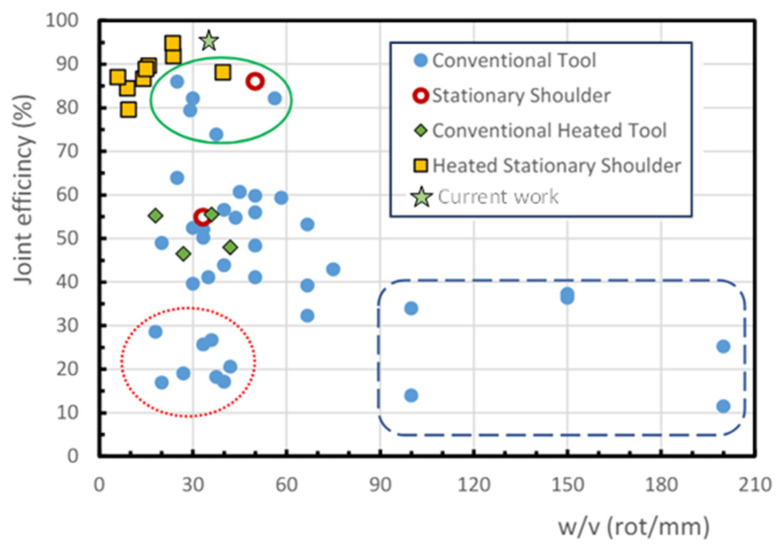
Effect of *w*/*v* ratio on *ξ* for different tool types and heat-assisted welding systems. Figure taken from reference [17].

In order to compare the results with data published in the literature, Figure 10 is taken from [17] and slightly modified to include the results obtained in the present research. In Figure 10, *ξ* of PP plates welded by means of FSW using different tools and *w*/*v* ratios is compared. It is worth pointing out that the highest *ξ* values are obtained using a heat-assisted device, stationary tool, and *w*/*v* between 5 and 40 rot/mm (see yellow squares and green star in Figure 10). These results confirm the high *ξ* that is obtained in the present investigation, which is denoted with a green star.

## 5. Conclusions

The effect of *w*, *v*, *T*, *W*, and crystallinity on the mechanical properties of polypropylene joints which are welded by means of a heat-assisted friction stir welding process using a non-rotational shoulder is studied in the present research work. The following conclusions are drawn from the investigation:

The highest efficiency value is obtained at *w* = 1500 rpm, *v* = 40 mm/min, and *T* = 80 °C, reaching a *ξ* = 94%.

The ANOVA shows that all processing parameters studied in the present research have a significant effect on the mechanical properties of the FSW joints, especially *w*.

The effect of the temperature on the microhardness and mechanical properties of PP FSW joints is beneficial because when increasing the PP temperature, the microhardness profile of the FSW joint becomes flatter (without pronounced hardness picks), similar to the virgin material, showing a higher ductility and *ξ* of the joint.

The temperature also has an important effect on the FSW joint appearance, considerably improving joint finish, texture, and defects such as voids that vanish along the weld line.

A correlation is evidenced between the parameter DZ/FA, the crystallinity of the SZ, and *ξ*. The results show a more amorphous SZ with a lower crystallinity for specimens with a higher *ξ* values.

## Figures and Tables

**Table 1:** Mechanical and physical properties of PP [8].

*E* GPa	*S*_y_ (MPa)	*S*_ut_ (MPa)	*T*_m_ (°C)	Density (g/cm^3^)	*T*_g_ (°C)
0.714	12.0–43.0	35.5 MPa	130–171	0.9705	−20

**Table 2:** Process parameter ranges.

Factor	Units	Low	High
*w*	rpm	1200	1500
*v*	mm/min	40	60
*T*	°C	60	80

**Table 3:** Tensile test results for the FSW samples.

Specimen	*ω* (rpm)	*v* (mm/min)	*T* (°C)	*S*_ut_ (MPa)	*ξ* (%)	*W*
1	1350	50	53	21.86	62%	36,450
2	1500	60	60	27.89	79%	37,500
3	1500	40	60	32.01	90%	56,250
4	1350	50	70	23.85	67%	36,450
5	1200	60	80	28.00	79%	24,000
6	1350	66	70	23.08	65%	27,614
7	1200	60	60	22.34	63%	24,000
8	1500	60	80	26.71	75%	37,500
9	1350	50	70	23.42	66%	36,450
10	1602	50	70	29.81	84%	51,328
11	1097	50	70	23.86	67%	24,068
12	1350	50	86	28.43	80%	36,450
13	1200	40	80	30.07	85%	36,000
14	1500	40	80	33.41	94%	56,250
15	1350	33	70	27.43	77%	55,227
16	1350	50	70	23.63	67%	36,450
17	1200	40	60	25.48	72%	36,000
18	1350	50	70	24.51	69%	36,450

**Table 4:** ANOVA for the tensile strength results.

Source	Square Sum	Freedom Degree	Mean Square	*F*-Ratio	*p*-Value
A:*w*	42.6583	1	42.6583	14.56	0.0051
B: *v*	39.9086	1	39.9086	13.62	0.0061
C: *T*	33.9085	1	33.9085	11.57	0.0093
AA	31.1614	1	31.1614	10.64	0.0115
AB	3.93401	1	3.93401	1.34	0.28
AC	12.5751	1	12.5751	4.29	0.072
BB	12.9278	1	12.9278	4.41	0.0689
BC	0.285013	1	0.285013	0.1	0.7631
CC	11.9522	1	11.9522	4.08	0.0781
Total Error	23.4405	8	2.93007	-	-
Total	197.791	17	-	-	-

**Table 5:** Ductile zone ratio and crystallinity.

Specimen	*DZ*/*FA*	Crystallinity	Reference
1	65.7	76.2	Present work
14	37.9	65.2	Present work
PP	-	36.4	[30]

## Data Availability

The original contributions presented in this study are included in the article. Further inquiries can be directed to the corresponding author(s).

## References

[1] Huang Y., Meng X., Xie Y., Wan L., Lv Z., Cao J., Feng J. (2018). Friction stir welding/processing of polymers and polymer matrix composites. Compos. Part A Appl. Sci. Manuf..

[2] Sahu S.K., Mishra D., Mahto R.P., Sharma V.M., Pal S.K., Pal K., Banerjee S., Dash P. (2018). Friction stir welding of polypropylene sheet. Eng. Sci. Technol. Int. J..

[3] Nandan R., DebRoy T., Bhadeshia H. (2008). Recent advances in friction-stir welding process, weldment structure and properties. Prog. Mater. Sci..

[4] Mishra R.S., Ma Z.Y. (2005). Friction stir welding and processing. Mater. Sci. Eng. R Rep..

[5] Lohwasser D., Zhan C. (2009). Friction Stir Welding: From Basics to Applications.

[6] Ribeiro L., Pinto G.F., Baptista A., Monteiro J. (2023). Hydrogen Electrical Vehicles.

[7] Maddah H.A. (2016). Polypropylene as a Promising Plastic: A Review. Prog. Mater. Sci..

[8] Campbell F. (2004). Manufacturing Processes for Advanced Composites.

[9] McCarville D.A., Schaefer H.A. (2001). Composites.

[10] Leskovics K., Kollár M., Bárczy P. (2006). A study of structure and mechanical properties of welded joints in polyethylene pipes, Composites. Mater. Sci. Eng. A.

[11] Payganeh G.H., Arab N.M., Asl Y.D., Ghasemi F.A., Boroujeni M.S. (2011). Effects of friction stir welding process parameters on appearance and strength of polypropylene composite welds. Int. J. Phys. Sci..

[12] Kusharjanta B., Raharjo W.P., Triyono T. (2016). Temperature Comparison of Initial, Middle and Final Point of Polypropylene Friction Stir Welded.

[13] Mirabzadeh R., Parvaneh V., Ehsani A. (2021). Experimental and numerical investigation of the generated heat in polypropylene sheet joints using friction stir welding (FSW). Int. J. Mater. Form..

[14] Stadler G.R., Szebényi G., Horváth R. (2023). Investigation of Weld Forces and Strength of Friction Stir Welded Polypropylene Period. Polytech. Mech. Eng..

[15] Reddy V.J., Rajasekhar A., Krishnaiah A. (2024). Impact of Process Parameters on the Mechanical Properties of Friction Stir Welded Joints on Polypropylene Sheets. Int. J. Mech. Eng..

[16] Kumar R., Singh R., Ahuja I., Penna R., Feo L. (2018). Weldability of thermoplastic materials for friction stir welding–A state of art review and future applications. Compos. Part B Eng..

[17] Pereira M., Amaro A., Reis P., Loureiro A. (2021). Effect of Friction Stir Welding Techniques and Parameters on Polymers Joint Efficiency–A Critical Review. Polymers.

[18] Moreno-Moreno M., Romero Y., Zambrano H.R., Restrepo C., Afonso C., Unfried J. (2018). Mechanical and thermal properties of friction stir welded joints of high density polyethylene using a non-rotational shoulder tool. Int. J. Adv. Manuf. Technol..

[19] Kiss Z., Czigány T. (2007). Applicability of friction stir welding in polymeric materials. Period. Polytech. Mech. Eng..

[20] Azarsa E., Mostafapour A. (2014). Experimental investigation on flexural behavior of friction stir welded high density polyethylene sheets. J. Manuf. Process..

[21] Banjare P.N., Sahlot P., Arora A. (2017). An assisted heating tool design for FSW of thermoplastics. J. Mater. Process. Technol..

[22] Panneerselvam K., Lenin K. (2013). Effects and defects of the polypropylene plate for different parameters in friction stir welding process. Int. J. Res. Eng. Technol..

[23] Jaiganesh V., Balaiya M., Elamparithy G. (2014). Optimization of Process Parameters on Friction Stir Welding of High Density Polypropylene Plate. Procedia Eng..

[24] Montgomery Douglas C. (2017). Design and Analysis of Experiments.

[25] (2003). Standard Test Method for Tensile Properties of Plastics.

[26] (2017). Standard Test Method for Microindentation Hardness of Materials.

[27] Kong Y., Hay J.N. (2002). The measurement of the crystallinity of polymers by DSC. Polymer.

[28] Noor Hasanah T.I.T., Wijeyesekera D.C., bin Bakar I., Saidin W. (2013). New lightweight construction material: Cellular mat using recycled plastic. Key Eng. Mater..

[29] Jones H., McClements J., Ray D., Hindle C.S., Kalloudis M., Koutsos V. (2023). Thermomechanical Properties of Virgin and Recycled Polypropylene—High-Density Polyethylene Blends. Polymer.

[30] Dodge Y. (2008). Least Significant Difference Test. The Concise Encyclopedia of Statistics.

[31] Razavi M., Cheng S., Huang D., Zhang S., Wang S.Q. (2020). Crazing and yielding in glassy polymers of high molecular weight. Polymer.

[32] Bowden P.B., Haward R.N. (1973). The Yield Behaviour of Glassy Polymers. The Physics of Glassy Polymers.

[33] Sichina W. (2000). Measurement of Percent Crystallinity of Thermoplastics.

[34] Butylina S., Martikka O., Kärki T. (2020). Physical and mechanical properties of wood-polypropylene composites made with virgin and/or recycled polypropylene. Polym. Plast. Technol. Eng..

[35] Nath R.K., Jha V., Maji P., Barma J.D. (2021). A novel double-side welding approach for friction stir welding of polypropylene plate. Int. J. Adv. Manuf. Technol..

[36] Nath R.K., Maji P., Barma J.D. (2019). Development of a Self-Heated Friction Stir Welding tool for welding of polypropylene sheets. J. Braz. Soc. Mech. Sci. Eng..

[37] Tan C.J., Andriyana A., Ang B.C., Wong D. (2020). Mechanical deformation and fracture mechanisms of polymeric fibres from the perspective of fractography–A review. Eur. Polym. J..

[38] Kusharjanta B., Soenoko R., Purnowidodo A., Irawan Y.S. (2022). Analysis of tensile strength, crystallinity, crystallite size, and thermal stability of polypropylene joined by friction stir welding. J. Appl. Eng. Sci..

[39] Li J., Zhu Z., Li T., Peng X., Jiang S., Turng L.S. (2020). Quantification of the Young’s modulus for poly-propylene: Influence of initial crystallinity and service temperature. J. Appl. Polym. Sci..

